# Hydroxyproline-Modified Chitosan-Based Hydrogel Dressing Incorporated with Epigallocatechin-3-Gallate Promotes Wound Healing Through Immunomodulation

**DOI:** 10.3390/gels11090732

**Published:** 2025-09-11

**Authors:** Peng Ding, Yanfang Sun, Guohua Jiang, Lei Nie

**Affiliations:** 1 School of Life Science, Xinyang Normal University, Xinyang 464000, China; dingzhiyu120@163.com; 2 College of Life Sciences and Medicine, Zhejiang Sci-Tech University, Hangzhou 310018, China; katherineyfs@zstu.edu.cn; 3 International Scientific and Technological Cooperation Base of Intelligent Biomaterials and Functional Fibers of Zhejiang Province, Zhejiang Sci-Tech University, Hangzhou 310018, China; ghjiang_cn@zstu.edu.cn; 4 School of Materials Science and Engineering, Zhejiang Sci-Tech University, Hangzhou 310018, China

**Keywords:** immunoregulation, anti-inflammation, wound healing, polysaccharide, EGCG

## Abstract

Immunoregulation is an emerging treatment strategy to promote wound healing by modulating the local immune system at the wound site. In this study, an extracellular matrix biomimetic and polysaccharide-based hydrogel was engineered to regulate the wound immune environment through Michael-type addition between maleimidyl pullulan and chitosan modified with hydroxyproline. The proposed hydrogel exhibited favorable injectable and self-healing properties, which facilitated the full coverage of irregularly shaped wounds. A natural polyphenol, epigallocatechin-3-gallate (EGCG), was incorporated into hydrogels, which thereby exhibited excellent biocompatibility, good reactive oxygen species (ROS) scavenging ability, anti-inflammatory activity, and antibacterial properties against *S. aureus* and *E. coli*. Furthermore, evaluations of a full-thickness skin defect mice model showed that the hydrogel with EGCG effectively alleviated the inflammatory response by reducing pro-inflammatory cellular infiltration and down-regulating the inflammatory cytokine TNF-α, while up-regulating anti-inflammatory cytokine IL-10. Notably, a faster wound healing rate was also achieved by the better promotion effect of the hydrogel on increasing the formation of re-epithelialization, granulation tissue generation, collagen deposition, and angiogenesis. Therefore, our immunoregulatory strategy showed great potential in the design of biomaterials for wound management.

## 1. Introduction

As the outer barrier of the body, the skin has evolved a set of complex and efficient immunoregulation mechanisms to maximally restore the tissue function and integrity when damaged [1]. Cells of the immune system are important participants in the wound repair process, in which skin injuries, if not treated promptly and effectively, are susceptible to excess oxidative stress arising from ROS and bacterial infection [2,3,4,5]. This can result in a sustained influx of pro-inflammatory cells, primarily composed of neutrophils and macrophages, at the wound site, which then promotes the excessive secretion of inflammatory cytokines and the expression of proteases [6,7]. Meanwhile, dermal reconstitution and blood vessel growth were significantly hindered by the high wound protease levels, which not only destroyed the local extracellular matrix, but also degraded growth factors [8,9]. These resulting consequences would prolong the transformation of wound from inflammation to proliferation, thus delaying the formation of new healthy tissues [10,11]. Therefore, it is of clinical importance to engineer novel wound dressings with the capacity to prevent bacterial invasion, scavenge ROS, and alleviate the inflammatory response and oxidative stress at the wound site, which expedites wound healing quickly and effectively. Compared with the traditional gauze and bandages dressings with low bioactivity and degradation [12,13], hydrogel has showed unparalleled advantages as potential candidates for wound dressings not only because of their biocompatibility and biodegradability, but also due to their customizable multifunctionalities (e.g., anti-inflammatory, antioxidant, and antibacterial properties) and tailored mechanical properties [14,15,16,17].

Apart from satisfactory antibacterial and antioxidant abilities of biomaterials, a suitable immune microenvironment is essential after biomaterial imposition for wound healing, which influences numerous physiological processes, including angiogenesis, re-epithelialization, and tissue remodeling [18,19]. An uncontrolled inflammatory reaction to implants may result in foreign body reactions, such as fiber encapsulation and implant defeat, ultimately inhibiting wound healing [20]. Thus, the strength and duration of inflammation would eventually decide the biocompatibility and dependability of the biomaterial implanted in body. Macrophages, as critical regulatory cells in the immune microenvironment, exhibit dynamic changes in phenotypes and functions during tissue regeneration [21,22,23]. M1-type macrophages, observed mainly in the initial tissue injury response, increase the expression of pro-inflammatory cytokines to promote innate immunity and wound debridement. However, persistent inflammatory response could prolong the wound healing process. M2-type macrophages produce anti-inflammatory cytokines and uptake pathogens, which help to resolve inflammation and accelerate tissue formation and remodeling [24,25]. Therefore, the convertible transformation of macrophages from M1-type to M2-type dominates the wound healing process. The active tuning of macrophage polarization has been considered an effective design strategy for biomaterials to exploit the host immune response, thus enhancing biomaterial-tissue incorporation and tissue repair.

Macrophages could also incisively sense immune signals at the wound site where biomaterials were implanted and accordingly showed different phenotypes. For instance, the polarization of macrophages can be regulated not only by the physicochemical properties of biomaterials, such as hydrophilicity and hydrophobicity [26], surface softness and hardness [27], and the components of materials [28], but also by the active compounds loaded in the biomaterials such as cytokines [29], inorganic metal ions [30], plant-derived drugs [31], and so on. These findings suggest that the immune response at the wound site can be modulated by tailoring the physicochemical properties of implanted biomaterials, thereby regulating macrophage polarization and promoting the transition from the inflammation to proliferation in wound repair process.

Natural polysaccharides, such as chitosan, hyaluronic acid, and pullulan, have been frequently used in hydrogel-based wound dressings due to their intrinsic biocompatibility and physicochemical and biological properties similar to those of the extracellular matrix [32,33], thereby providing a new and healthy niche for cell infiltration and proliferation. Epigallocatechin-3-gallate (EGCG) is a well-known natural polyphenol extracted from green tea and exhibits cardiovascular protection, anti-oxidative and antibacterial properties [34,35,36]. However, the rapid metabolism and light sensitivity of EGCG limit its bioavailability, while the direct administration of EGCG at the wound site may induce toxicity due to its high local concentration, thus leading to an ineffective therapeutic effect [37,38]. Therefore, EGCG was generally combined with nanoparticles or encapsulated into hydrogels to enhance its stability and bioavailability [39,40]. Furthermore, it also inspires us to investigate the therapeutic effects of EGCG on wound healing, especially its influences on the local immune microenvironment and angiogenesis at the wound site.

**Scheme 1:**
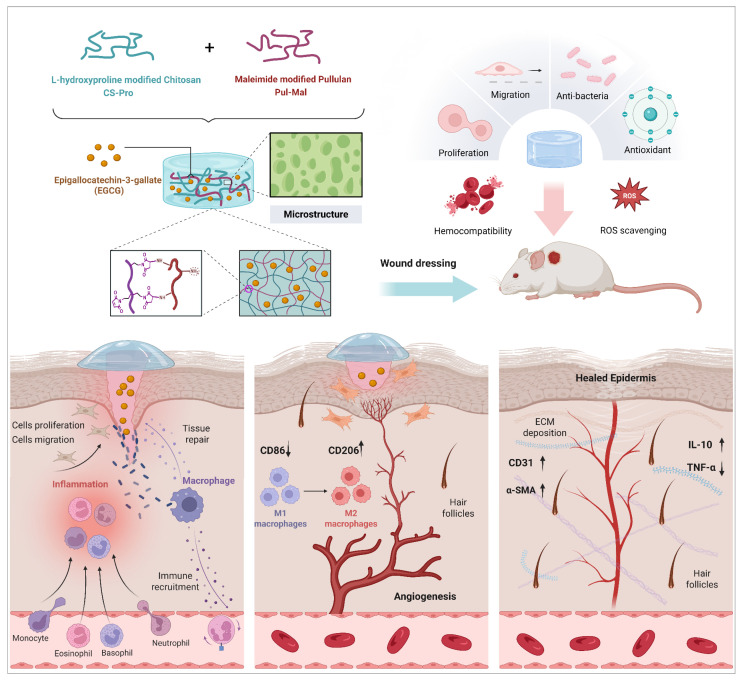
Schematic illustration of the preparation of polysaccharides-based hydrogel with multifunctional properties and its therapeutic effect for skin regeneration.

In this paper, we developed an extracellular matrix biomimetic (ECM) and polysaccharide-based hydrogel dressing incorporated with EGCG to study its influences on the regulation of immune microenvironment at the wound site (Scheme 1). The Pullulan and chitosan were chosen as hydrogel backbones for their excellent bioactivity in immune regulation and tissue repair. Hydroxyproline was an amino acid found in collagen elastin that was an important component of ECM and could improve wound healing by promoting the expression of new collagen and the repair of damaged epithelial structures [41,42]. Then, it was grafted to chitosan to recapitulate the glycoprotein components in ECM [43,44,45] and improve the solubility of chitosan in water and its compatibility. Subsequently, hydroxyproline-modified chitosan reacted with 3-maleimidopropionic acid-modified pullulan through Michael-type addition to engineer the composite hydrogel. It not only exhibited injectability, self-healing, and tailored mechanical properties, but also excellent biocompatibility, antioxidant, and antibacterial properties. Notably, when evaluated in a full-thickness skin defect mice model, it can obviously reduce wound inflammation and accelerate wound healing by increasing angiogenesis, collagen deposition, and granulation tissue formation. Taking these advantages into account, our immunomodulation-based strategy has shown great potential for clinical application.

## 2. Results and Discussion

### 2.1. Synthesis of Pul-Mal and CS-Pro

**Figure 1:**
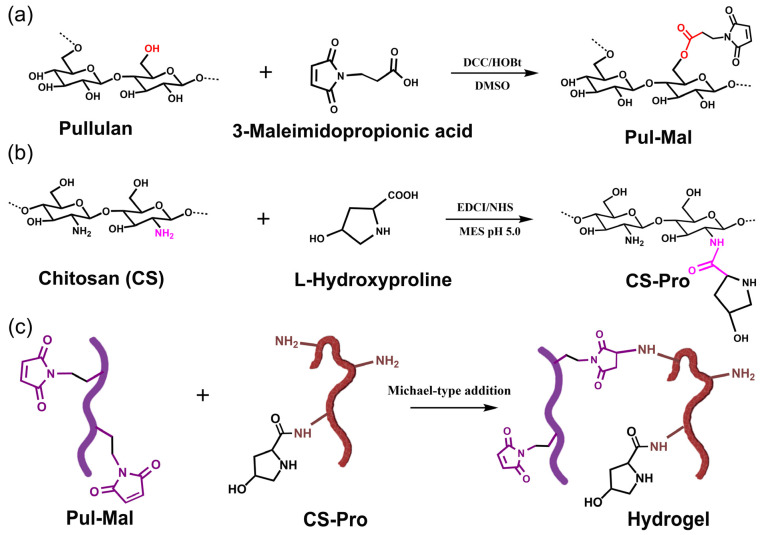
Synthesis process of Pul-Mal (**a**) and (**b**) CS-Pro. (**c**) Illustration of Michael-type addition between Pul-Mal and CS-Pro.

Inspired by the proteoglycan in ECM, chitosan was modified with L-hydroxyproline to recapitulate the glycoprotein components in ECM. Pul-Mal was prepared through esterification between pullulan and 3-maleimidopropionic acid (Figure 1a). Then the multifunctional hydrogel was formed by homogeneously mixing Pul-Mal and CS-Pro containing different concentrations of EGCG through Michael-type addition (Figure 1c) [46]. The successful synthesis of Pul-Mal and CS-Pro was determined by ^1^H NMR and FT-IR spectra.

**Figure 2:**
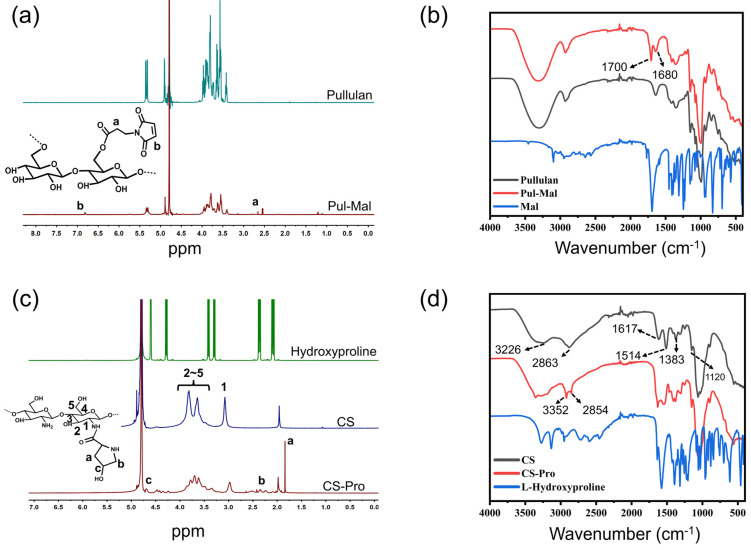
(**a**) ^1^H NMR and (**b**) FT-IR spectra of Pullulan, Pul-Mal and Mal. (**c**) ^1^H NMR and (**d**) FT-IR spectra of CS, CS-Pro, and L-hydroxyproline.

As shown in Figure 2a, compared with the spectra of Pullulan, the new characteristic absorption peak at 6.84 ppm in Pul-Mal was ascribed to the ^1^H proton peak of -CH=CH- in the maleimide group [47], while the peaks at 2.75~2.83 ppm were assigned to the methylene (-CH_2_-CH_2_-) of maleimide adjacent to the amide bond. Moreover, the lyophilized Pul-Mal showed new absorbance bands at 1700 cm^−1^ and 1680 cm^−1^ in the FT-IR spectrum, corresponding to the stretching vibration of -C=O and -C=C- from the maleimide group, respectively (Figure 2b). The above results confirmed the successful grafting of maleimide to pullulan. The degree of substitution (DS) of 3-maleimidorpopionic acid in Pul-Mal was calculated by the ratio of the peak area corresponding to the hydrogen atom of the -C=C bond in the maleimide groups (δ = 6.84 ppm) to the peak area corresponding to the hydrogen atom (δ = 5.24~5.44 ppm) on the main chain of pullulan, and the DS was about 6%.

**Figure 3:**
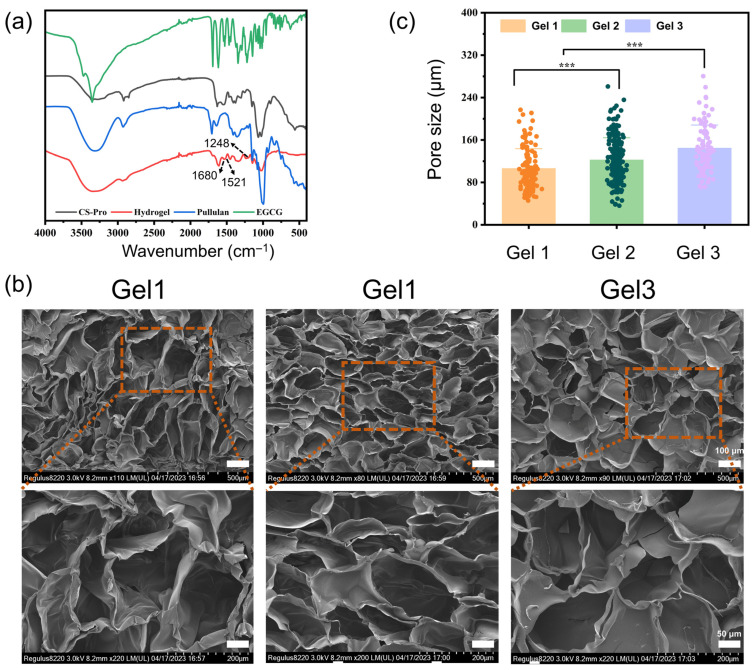
(**a**) FT-IR spectra of EGCG, Pullulan, CS-Pro and Hydrogel. (**b**) SEM images and pore size (**c**) of hydrogels (*** *p* < 0.001).

As shown in Figure 2c, the characteristic peaks at 3.07 ppm and 3.64~3.83 ppm corresponded to ^1^H protons at positions 1, 2, 3, 4, and 5 of the CS, respectively [48]. Compared with CS, the peaks at 1.82 ppm, 2.35 ppm and 4.62 ppm in the spectra of CS-Pro corresponded to the ^1^H absorption peaks at position “a”, “b” and “c” of L-hydroxyproline, respectively. In the FT-IR spectra of Chitosan (Figure 2d), the peak at 3226 cm^−1^ and 2863 cm^−1^ was attributed to the stretching vibration of -OH bond and that of C-H, respectively. In addition, the adsorption peak at 1615 cm^−1^, 1514 cm^−1^, 1383 cm^−1^ and 1120 cm^−1^ was assigned for the -C=O stretching vibration of amide I band, bending vibration of the N-H (amide II band), OH bending and anti-symmetric stretching vibration of the C-O-C bridge, respectively [49,50]. In the spectra of CS-Pro, the adsorption peaks at 3352 cm^−1^ and 2852 cm^−1^ correspond to the stretching vibrations of N-H and -CH_2_ in hydroxyproline, respectively. Moreover, the intensity of the -C=O stretching vibration increased, while that of the N-H bending decreased, which could be attributed to the formation of an amide bond between hydroxyproline and chitosan. These results confirmed that the hydroxyproline was successfully grafted to the chitosan. Through ^1^H NMR spectrum of CS-Pro, the DS of hydroxyproline in CS-Pro was calculated from the peak area corresponding to the hydrogen atom at the “c” peak of hydroxyproline (δ = 4.68 ppm) to the peak area corresponding to the hydrogen atom at the chitosan backbone (“1”, δ = 2.98 ppm). The DS of hydroxyproline was calculated to be about 53%. As seen in Figure 3a, the successful preparation of hydrogel was also characterized by FT-IR. Compared with Pul-Mal, the hydrogel had a weak peak at 1680 cm^−1^, indicating that the -C=C- group in 3-meleimidopropionic acid was consumed by Michael addition. Meanwhile, there was e new peak at 1248 cm^−1^, which was ascribed to the stretching vibration of C-N due to the reaction between Pul-Mal and CS-Pro. Moreover, the new peak at 1521 cm^−1^ was ascribed to deformation vibration of the benzene form EGCG, conforming that EGCG was encapsulated into hydrogels.

### 2.2. Physicochemical Properties of the Composite Hydrogels

**Figure 4:**
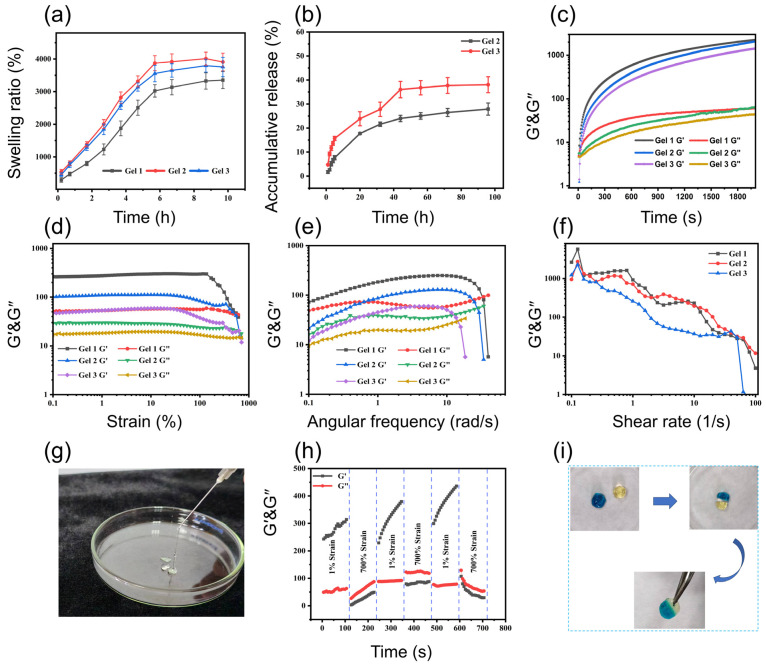
(**a**) Swelling ratio of hydrogels. (**b**) Release profile of EGCG in the composite hydrogels. (**c**) Time sweep, (**d**) Strain sweep, (**e**) Frequency sweep, (**f**) Viscosity of hydrogels. (**g**) Extrusion of Gel 1 through the needle. (**h**) Continuous time sweep of Gel 1 with alternate strain for three cycles (low strain at 1% for 100s and high strain at 700% for 100 s). (**i**) Macroscopical self-healing property of Gel 1.

Furthermore, the surface morphology of the hydrogels was characterized through SEM. The images obtained in Figure 3b indicated that the prepared hydrogels possessed a three-dimensional porous structure, which helped to improve water absorption, drug release, nutrient transportation, cell migration, and proliferation [51]. As shown in Figure 3c, the pore diameter of Gel 1, Gel 2, and Gel 3 was 105.9 ± 27.7 μm, 121 ± 42.4 μm, and 144.2 ± 44.1 μm, respectively. This result showed that pore size of hydrogels increased with the gradual increase in EGCG concentration, which might be because the addition of EGCG waken the cross intensity of hydrogels. Furthermore, an appropriate swelling ratio was necessary for wound dressings to maintain a moist environment in the wound, which would promote wound healing. Interestingly, the addition of EGCG also influence the swelling ratio of hydrogels. As shown in Figure 4a, swelling ratio of hydrogels in the equilibrium stage first increased and then decreased. It showed that the composite hydrogels exhibited a strong water absorption capacity, with swelling ratios of 3353%, 3908%, and 3755% after immersion in PBS solution, respectively. That suggested these hydrogels possessed excellent ability to absorb tissue exudate and maintained a most environment, which benefited to reduce infection and accelerate the wound healing. To attain effective therapeutic outcomes, a sustained release of EGCG was preferred at the wound site. Then the in vitro release behaviors of EGCG in the hydrogels were investigated. As shown in Figure 4b, Gel 2 and Gel 3 exhibited a continuous release profile without significant burst release, and the release rate in 96 h for Gel 2 and Gel 3 was 27% and 38%, respectively. The accumulative release rate of Gel 2 was lower than that of Gel 3. That was because Gel 2 had a more compact network structure than Gel 3.

Hydrogel-based wound dressings with suitable mechanical properties are highly appreciated when used in medical applications. Herein, the rheological properties of hydrogels were characterized by a rheometer. As shown in Figure 4c, the storage modulus (G’) and loss modulus (G”) in the time sweep test increased along with time, and the value of G’ was still larger than G”, indicating the formation of a gel. It was worth noting that the value of G’ decreased with the gradual growth of EGCG concentration, indicating that the addition of EGCG might weaken the hydrogel network. That was probably due to the hindering effect of EGCG to the cross-link of hydrogel backbones. When subjected to the strain sweep, G′ kept relatively stable during the linear viscoelastic regime (Figure 4d). As the strain continuously rose, the G’ gradually decreased until a certain point, where G” was larger than G’, indicating the collapse of the hydrogel structure. In 0.1~10 Hz frequency sweep range (Figure 4e), G’ was larger than G”, exhibiting the stable structure and elasticity of hydrogel. However, when the frequency increased, G’ gradually decreased to the crossing point where G” exceeded G’, denoting the disruption of hydrogel network. In brief, both strain sweep and frequency sweep results showed that the addition of EGCG weakened the mechanical properties. In Figure 4f, the viscosity of hydrogels decreased along with the increase in shear rate from 1 to 100 s^−1^, showing that hydrogels possessed the shear-thinning property. This would allow the hydrogel to be injected directly and fully cover the irregularly shaped wound site (Figure 4g). Moreover, when applied in medical applications, the structure of hydrogels is inevitably damaged or deformed by external forces, thus compromising their therapeutic efficacy. Therefore, hydrogels with self-healing properties can restore their network structure through reversible chemical bonds, thus extending their usage lifespan. In this study, the step strain test was conducted to evaluate the rheological recovery behavior of hydrogel (Figure 4h). Taking Gel 1 for example, under 1% strain, G’ was larger than G”, indicating the formation of a gel network. When under high strain (700%), G” was larger than G’, and the network structure of hydrogel was destroyed. Interestingly, when the strain was adjusted to low strain (1%), G’ was again larger than G” during the test, meaning the restoration of the hydrogel network structure. Figure 4i exhibited the macroscopic self-healing test of Gel 1. Two pieces of hydrogels with different colors could integrate after contacting for 1 h owing to a Michael-type addition between the unreacted amine group from chitosan and the maleimide group from Pul-Mal.

### 2.3. Antibacterial Properties of Hydrogels

**Figure 5:**
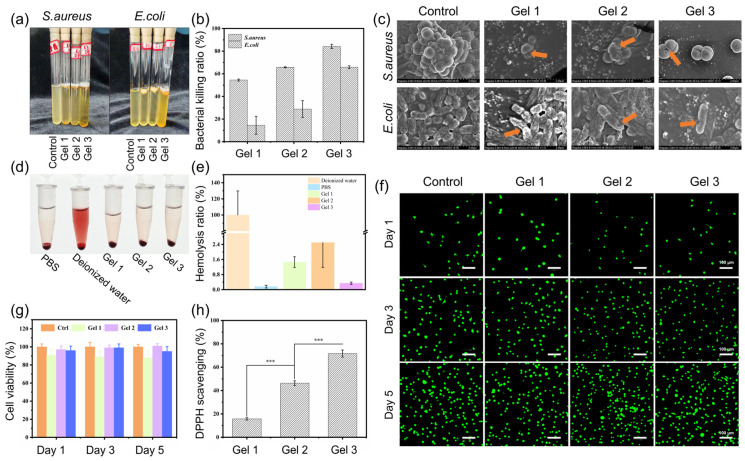
(**a**) Photographs and (**b**) bacterial killing ratios of hydrogels against *S. aureus* and *E. coli*. (**c**) SEM images of *S. aureus* and *E. coli* after being cultured with hydrogels. Orange arrows indicated the damaged cell wall of bacteria. (**d**) Hemolytic photographs and (**e**) Statistical hemolysis ratios of hydrogels. (**f**) Images of L929 cells after being cultured with hydrogel extract for 1, 3, and 5 days by Live/Dead fluorescence staining. (**g**) Survival rates of L929 cells incubated with hydrogel for 1, 3, and 5 days via CCK-8 assay. (**h**) DPPH scavenging efficiency of hydrogels (*** *p* < 0.001).

Microorganism invasion can result in an adverse and persistent inflammatory response, in which macrophages and neutrophils accumulate at the wound site and then secrete inflammatory cytokines, activate the production of metalloproteinases, and destroy the newly formed and regenerated tissue [52,53] thus hindering tissue repair and wound healing [54,55]. Therefore, the antibacterial activities of hydrogels were investigated against *E. coli* and *S. aureus*. In Figure 5a,b, the hydrogels showed EGCG content-dependent antibacterial activity. The kill ratio of Gel 1, Gel 2, and Gel 3 for *E. coli* was 14.5%, 28.9% and 65.8%, while that for *S. aureus* was 54.5%, 65.7% and 84.1%, respectively. Moreover, as shown in Figure 5c, compared with the control group, SEM images revealed that the cell wall of bacteria cultured with hydrogels was significantly damaged, confirming the outstanding inhibiting efficiency of hydrogels, which was attributed to the synergistic effect of EGCG and chitosan.

### 2.4. Biocompatibility of Hydrogels

The biocompatibility of biomaterials when used in vivo is a widely concerned issue. Hence, a hemolysis test was conducted to evaluate the blood compatibility of hydrogels. In the hemolysis evaluation, deionized water was considered as the positive control group, while PBS buffer solution served as the negative control group. As shown in Figure 5d, compared with the positive control group, the supernatant in the hydrogel treated groups was clear and transparent, with no blood cell precipitation at the bottom. The hemolysis ratios of all groups (Figure 5e) were lower than the international standard of 5% [56].

Furthermore, the biocompatibility of the prepared hydrogels was evaluated through coculture between L929 cells and hydrogel extract. The results of Live/Dead staining in Figure 5f exhibited that green fluorescence labeling live cells, was observed in all hydrogel groups. After 5 days’ incubation, compared with the control group, the hydrogel groups with EGCG showed no large area of cell death. The cell viability rates in all groups were higher than 70% (Figure 5g), which met the international biomaterial standards (ISO 10993, 2009) [15]. These results exhibited that the prepared composite hydrogels possessed excellent cytocompatibility.

### 2.5. Antioxidant and Anti-Inflammatory Activities

Excessive production of ROS can disrupt the intracellular balance between oxidation and antioxidation, leading to oxidative damage in the wound environment and thereby aggravating the inflammatory response and delaying tissue repair and wound healing [23,57]. Herein, hydrogel dressings with strong ROS scavenging ability are beneficial for adjusting the wound microenvironment and promoting wound healing. The efficiency of scavenging DPPH was investigated to assess the antioxidative activity of the hydrogels. In Figure 5h, the DPPH scavenging rates of the hydrogels increased with the gradual increase in EGCG concentration, likely due to the multiple phenol groups of EGCG.

**Figure 6:**
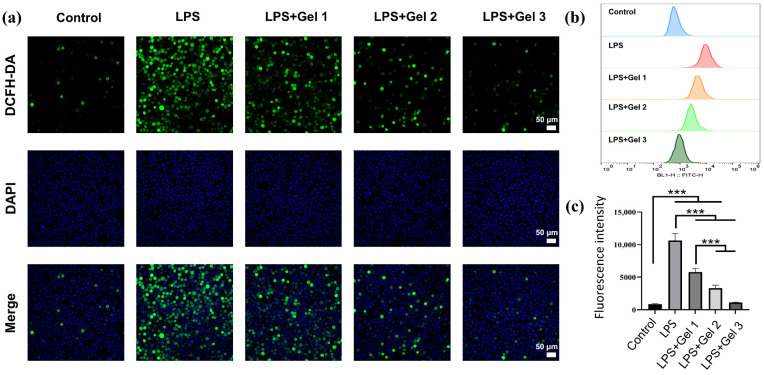
Intracellular antioxidant capacity of hydrogels. (**a**) Fluorescence images of RAW 264.7 cells stained with DCFH-DA after different treatments. (**b**) Flow cytometry and (**c**) quantitative analysis of intracellular ROS level of RAW264.7 cells after different treatments. *** *p* ≤ 0.001.

Furthermore, the intracellular ROS levels of macrophages (RAW 264.7) cells in response to lipopolysaccharide (LPS) treatment were detected by DCFH-DA staining for evaluating the scavenging efficiency of our hydrogels on ROS. DCFH-DA could enter the macrophages and easily oxidized by intracellular ROS into a compound with high fluorescence [16]. In Figure 6a, the control group presented no obvious green fluorescence, while the LPS-treated group showed strong green fluorescence. That indicated LPS induced macrophages to produce excessive ROS. However, as shown in Figure 6b,c, the green fluorescence of the hydrogel groups was significantly reduced compared with the LPS-treated group. These results indicated that the composite hydrogels with EGCG had a favorable antioxidative ability and possessed great potential to clear free radicals, which was beneficial for relieving inflammatory responses at the wound site.

### 2.6. In Vivo Wound Closure and Healing

**Figure 7:**
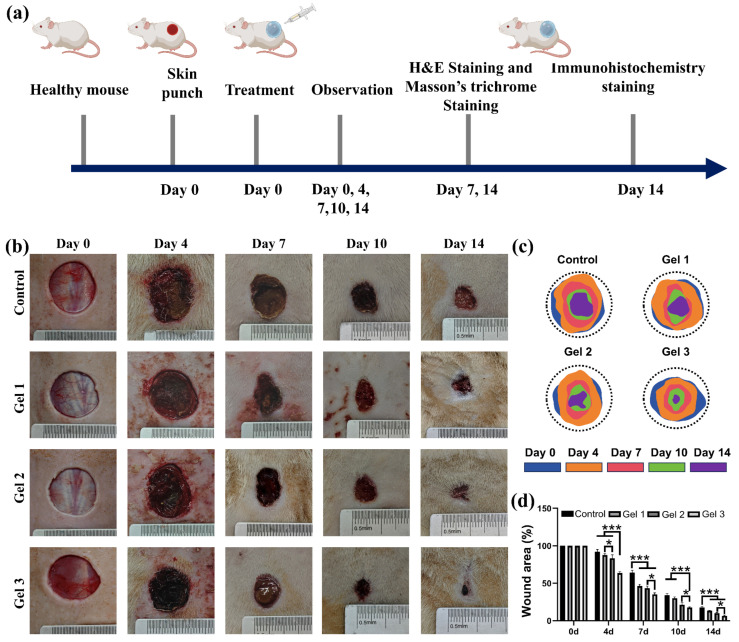
In vivo experiments on wound healing in the full-thickness mouse skin defect model. (**a**) Schematic diagram of the experimental schedule. (**b**) Photographs of representative repaired wounds in different groups at days 0, 4, 7, 10, and 14, respectively. (**c**) The schematic diagram of wound healing process subjected to different treatments. (**d**) Quantitative analysis of the wound remaining area in different groups (* *p* < 0.05 and *** *p* < 0.001).

A full-thickness skin defect mice model was used to evaluate the promoting effect of hydrogels on wound healing. As shown in Figure 7a–c, the wound healing process of full-thickness skin defect mice model was photographed on day 0, 4, 7, 10, and 14. On the 7th day after wound formation and hydrogel treatment, the control group had the highest wound area, at 64% (Figure 7d). However, compared with the control group, Gel 1 and Gel 2 significantly reduced the wound area, with the increase of 18% and 21% in closure percentage, respectively. Notably, Gel 3 achieved a healing rate of 65%, demonstrating the most effective therapeutic effects. On day 14, the wound area in all groups shrank, while Gel 3 exhibited the best performance with a wound closure percentage of 93%. Nevertheless, the wound regions were still observed in other groups, while the wound areas of the control group, Gel 1, and Gel 2 were 18%, 14%, and 10%, respectively. These results indicated that EGCG from the hydrogel significantly promoted wound healing.

### 2.7. Histological and Immunohistochemical Evaluation of Wound Regeneration

**Figure 8:**
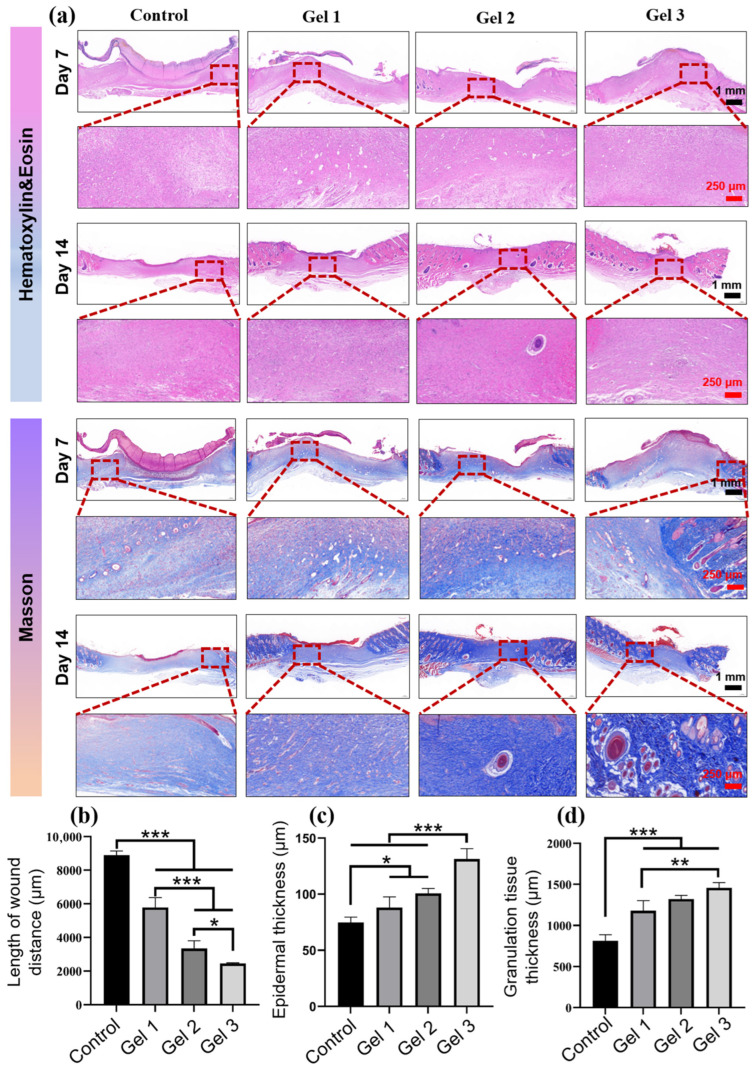
Therapeutic effects of hydrogels on wound healing. (**a**) Representative images of H&E and Masson histological staining of wound sections on day 7 and day 14. Quantitative analysis of the length of wound distance (**b**), epithelial thickness (**c**), and granulation tissue thickness (**d**) on day 14. * *p* < 0.05, ** *p* < 0.01, and *** *p* < 0.001.

To assess wound healing equality, tissue sections from the wound site on days 7 and 14 were excised for H&E staining and Masson staining. Wound closure was a key indicator for evaluating the wound healing process. The length of wound tissue and the thickness of epidermis and granulation tissue on day 14 were measured from the images of H&E (Figure 8a). On day 14, compared with other groups, Gel 3 showed superior healing efficiency in reducing wound length, with a wound distance of 2456 μm (Figure 8b). In contrast, the control group, Gel 1, and Gel 2 had a wound distance of 8892, 5790, and 3342 μm, respectively. Moreover, the thickness of epidermis and granulation tissue was also an important indicator for wound healing. On day 7, the granulation tissue in all groups was loose and incomplete, as shown in Figure 8a. However, on day 14, the Gel 3 group had a much thicker layer of epidermis and granulation tissue than the other groups. In addition, Masson staining was performed to assess the collagen deposition, which was colored blue. The hydrogel groups possessed the highest amount of collagen, as indicated by the images of the stained tissue, which showed a deeper blue color compared with the control group. These results suggested that the hydrogel exhibited excellent therapeutic effects in promoting wound healing, likely due to the antioxidative properties of EGCG and its ECM-mimicking structure.

**Figure 9:**
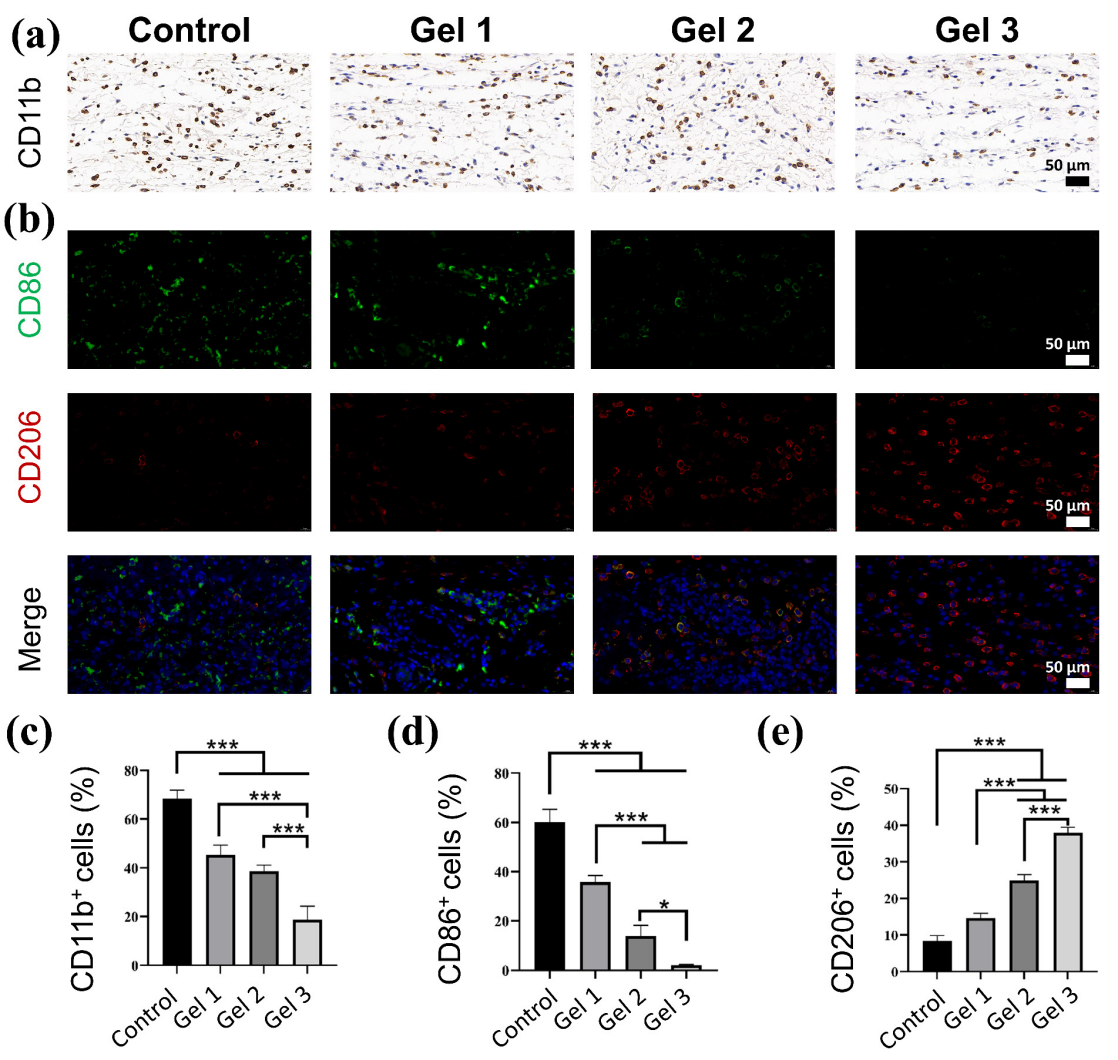
Immunofluorescent images of wound sections on 4th postoperative day. (**a**) Representative images and (**c**) quantitative analysis of CD11b staining. (**b**) Representative images and (**d**,**e**) quantitative analysis of CD86/CD206 immunofluorescence staining of wound sections. * *p* < 0.05, and *** *p* < 0.001.

It has been acknowledged that the wound inflammation microenvironment is of great importance in the wound healing process and tissue repair. Severe inflammation can recruit more monocytes and macrophages to accumulate in wounds, which further secrete inflammatory cytokines, promote the production of metalloproteinases, and impair wound healing, thus creating a vicious cycle [58,59]. To assess the degree of inflammatory response at the wound site, the infiltration of immune cells, including monocytes and macrophages, was quantified by CD11b staining. As shown in Figure 9a, the control group had the highest number of CD11b cell infiltration, and the infiltration rate of CD11b cells was 68%, indicating a serious inflammatory response, while that of Gel 1, Gel 2, and Gel 3 was, 45%, 39% and 19%, respectively. Among the various immune cells, macrophages play a crucial role in mediating the inflammatory response and are typically divided into two main types: pro-inflammatory M1 macrophages and anti-inflammatory M2 macrophages [60]. M1-type macrophages are a crucial indicator for the inflammatory response and produce substantial amounts of pro-inflammatory cytokines, facilitating the effective control and clearance of infections [61]. Meanwhile, M2-type macrophages are anti-inflammatory and possess the ability to upregulate anti-inflammatory cytokines that expedite tissue repair and wound healing [62,63]. Therefore, the M1-type and M2-type macrophages at the wound site were stained with CD86 (green) and CD206 (red) for visual observation, respectively. As presented in Figure 9b–d, the control group exhibited strong green fluorescence, suggesting a high infiltration rate of M1 macrophages and a serious inflammatory environment. In contrast, the number of M1 macrophages in the hydrogel groups decreased significantly along with the increase in EGCG concentration, implying a low degree of inflammatory response. It is noteworthy that the gradual decrease in M1 macrophages and increase in M2 macrophages, is a crucial event in the transition of wound healing from inflammation to the proliferation stage [64]. Interestingly, compared with the control group, a larger amount of M2 macrophages was observed in the hydrogel group with EGCG (Figure 9e). These results suggested that EGCG could effectively reduce the influx of immune cells and alleviate the inflammatory response by promoting the transition from the M1-type to the M2-type, subsequently facilitating wound repair. This was further confirmed by the expression of various cytokines at the wound site.

**Figure 10:**
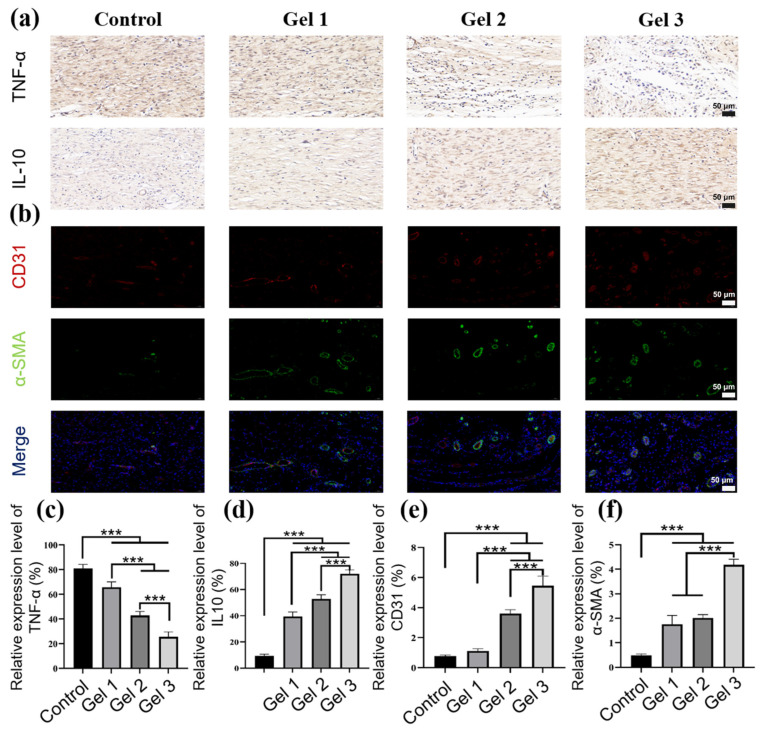
(**a**) Immunofluorescence images of TNF-α and IL-10. (**b**) Immunofluorescence images of CD31 and α-SMA. Quantitative statistics of the expression level of immunohistochemical TNF-α (**c**), IL-10 (**d**), CD31 (**e**), and α-SMA (**f**). *** *p* < 0.001.

As depicted in Figure 10a–c, the higher expression of TNF-α in the control group indicated a more serious inflammatory response than other groups. Meanwhile, a higher production of IL-10 in the hydrogel groups manifested a weaker inflammatory response, compared with the control group. These results suggested that EGCG, with its ROS scavenging ability, can attenuate the inflammatory response by downregulating TNF-α and upregulating IL-10, thereby benefiting reduction in the inflammatory response and accelerating wound healing. Additionally, vascularization is considered an important event in wound healing, as tissue regeneration relies on blood vessels to transport oxygen and nutrients [65,66]. Therefore, the expression levels of platelet endothelial cell adhesion molecule-1 (CD31) and α-smooth muscle actin (α-SMA) were assessed through immunofluorescence staining to evaluate neovascularization and maturation [67]. As shown in Figure 10b–f, all the hydrogel groups exhibited higher expression levels of CD31 and α-SMA than the control group, while the Gel 3 group showed the highest expression of CD31 and α-SMA, suggesting EGCG possessed better angiogenesis ability and could effectively drive wound repair.

## 3. Conclusions

In summary, a polysaccharide-based and ECM biomimetic composite hydrogel was engineered through Michael-type addition between maleimidyl pullulan and chitosan modified with hydroxyproline. The engineered hydrogels showed good viscoelasticity, self-healing properties, and ECM-mimicking structure. Natural polyphenol EGCG was loaded into the hydrogels, exhibiting a sustained release profile that exerted multiple synergistic effects, including antioxidative, antibacterial, and anti-inflammatory activities. Notably, when applied in a full-thickness mouse skin defect model, the sustained release of EGCG could regulate the inflammatory response at the wound site, facilitating the transition of macrophages from the M1 to the M2 phenotype, thereby allowing the wound healing process to transition smoothly from the inflammatory phase to the proliferative phase. Most importantly, the hydrogel-treated group achieved a faster wound healing rate by promoting re-epithelialization, granulation tissue generation, collagen deposition, and angiogenesis. Therefore, considering all the benefits, our proposed hydrogel dressing has great potential in clinical trials related to skin injury management.

## 4. Materials and Methods

### 4.1. Materials

Chitosan (CS with a degree of deacetylation of 90%, M_w_ ≈ 2 × 10^5^ Da), L-hydroxyproline (99%), Pullulan (M_w_ ≈ 5 × 10^5^ Da), 1-(3-dimethylaminopropyl)-3-ethylcarbodiimide hydrochloride (EDC·HCl, 98%), Epigallocatechin-3-gallate (EGCG, 98%), dimethyl sulfoxide (DMSO, >99.8%) 2-Morpholinoethanesulfonic Acid (MES, >99.5%), 1-hydroxybenzotriazole (HOBT, 99%), N-Hydroxysuccinimide (NHS, 98%), 3-maleimidopropionic acid (Mal, >97%), 2,2-diphenyl-1-(2,4,6-trinitrobenzene) hydrazine (DPPH, >98.5%), 2′,7′-dichlorodihydrofluorescein diacetate (DCFH-DA, 97%), dicyclohexylcarbodiimide (DCC, 99%), were purchased from Aladdin (Shanghai, China). All of the other commercial reagents and solvents were used without further purification.

### 4.2. Preparation of Pullulan Modified with Maleimide (Pul-Mal)

Pullulan modified with maleimide was synthesized according to the reported method with slight modifications [47]. 1 g of pullulan polysaccharide, 0.62 g of DCC, and 0.3 g of HOBT were dissolved in 30 mL of DMSO. 0.25 g of 3-maleimide propionic acid was dissolved in 10 mL of DMSO and then added into the aforementioned solution. The mixture reacted at 37 °C for 48 h and dialyzed in deionized water with a dialysis membrane (8000–14,000 Da, Yuanye, Shanghai, China) for 5 days. After freeze-drying, the product was stored in a refrigerator at −20 °C for further use.

### 4.3. Chitosan Functionalized with L-Hydroxyproline (CS-Pro)

Firstly, 2 g chitosan was dissolved in 200 mL 1% acetic acid aqueous solution. Then, 1.6 g of L-hydroxyproline, 1.6 g of NHS, and 2.8 g of EDC·HCl reacted in 50 mL of MES buffer (pH 5.0) for 30 min and then added into chitosan solution and reacted for 24 h. Finally, the mixed solution was dialyzed in water using a dialysis membrane (8000–14,000 Da, Yuanye, China) for 5 days, and then lyophilized to obtain the product.

### 4.4. Chemical Characterization of Synthesized Polymers

The chemical characterization of CS-Pro and Pul-Mal was studied by Fourier transform infrared spectroscopy (FT-IR) and nuclear magnetic resonance (^1^H NMR).

FT-IR spectra of CS-Pro, CS, pullulan, and Pul-Mal were characterized within the range between 4000 and 400 cm^−1^ on FTIR spectrometer (Thermofisher IS50, Thermo Fisher Scientific, Waltham, MA, USA) with a resolution of 4 cm^−1^.

Nuclear magnetic resonance (^1^H NMR): 20 mg CS-Pro, CS, pullulan, and Pul-Mal were dissolved in 700 μL D_2_O. The spectra were conducted by HNMR spectrometer at 600 MHz (JEOL ECZ600R/S3, JEOL, Tokyo, Japan).

### 4.5. Preparation of the Composite Hydrogels

0.06 g Pul-Mal was dissolved in 1 mL PBS (pH 7.4, 10 mM) to obtain 6% *w*/*v* Pul-Mal solution. A 3.5% *w*/*v* CS-Pro solution was prepared by dissolving 0.175 g CS-Pro in 5 mL of PBS. Subsequently, 0.05 g and 0.15 g of EGCG were added into the CS-Pro solution, respectively. Finally, the hydrogel was engineered by blending a 6% Pul-Mal solution and CS-Pro/EGCG solution in equal volumes at room temperature. The as-prepared hydrogels with 1% and 3% EGCG content were designated as Gel 2 and Gel 3, respectively. In the meantime, the hydrogel without EGCG served as the control group and was designated as Gel 1.

### 4.6. Characterization of Hydrogels

#### 4.6.1. Hydrogel Morphology

The prepared functional hydrogels were freeze-dried and then cut into slice samples. Subsequently, the platinum layer needs to be sprayed on the surface of the slices. Finally, the internal microstructure of the hydrogels was characterized using a scanning electron microscope (SEM) (Hitachi S-4800, Hitachi, Tokyo, Japan).

#### 4.6.2. Swelling Rate Test

The swelling rate test was carried out in deionized water at room temperature. The freeze-dried hydrogels were first weighed (*W_c_*) and then soaked in PBS solution. Then, the hydrogel was removed at a predetermined time interval, and then weighed (*W_s_*) after the elimination of the water on the surface of hydrogel. Finally, the swelling rate was determined through the below equation:

$$Swelling\;rate=\;\frac{W_{s}-W_{c}}{W_{c}}\;\times 100\%$$

#### 4.6.3. Release Profile of EGCG in Hydrogels

Firstly, EGCG solutions of 2 μg/mL, 5 μg/mL, 10 μg/mL, 20 μg/mL, 50 μg/mL and 100 μg/mL were prepared with deionized water, and the absorption value of different concentrations was measured by a UV-Vis spectrophotometer at 272 nm (PerkinElmer Lambda 950, PerkinElmer, Waltham, MA, USA) to make the standard curve of EGCG. Then, 2 mL of Gel 1, Gel 2 and Gel 3 was first prepared according to the preparation of the composite hydrogels described in Section 4.5. Then, the composite hydrogels were immersed in a conical beaker with 400 mL of deionized water at room temperature. 3 mL of hydrogel extract solution was collected at predetermined time intervals and measured using a UV-Vis spectrophotometer at 272 nm. After that, 3 mL of deionized water was added the conical beaker to make sure that the volume of water was still 400 mL. Finally, the release amount of EGCG was calculated according to the standard curve.

### 4.7. Rheological Analysis

A TA rheometer (DHR-2, TA Instruments, New Castle, DE, USA) in oscillation mode was used to characterize the Rheological properties of the hydrogels, which was described in a previous report [68]. All tests were conducted at 37 °C. Time sweep was conducted for 30 min at a frequency of 1 Hz and strain of 1%. The strain sweep was performed from 0.1% to 700% to determine the linear viscoelastic regime at a frequency of 1 Hz. A frequency sweep was conducted from 1 to 100 rad/s with a strain of 1%. The continuous step strain time sweep was performed to test the rheological recovery properties of the hydrogel at a frequency of 1 Hz. The viscosity of hydrogels was conducted at different shear rates (0.1–100 s^−1^). Two disk-shaped hydrogels were prepared with a diameter of 8 mm and a thickness of 2 mm, while one of the hydrogels was stained with methylene blue. Then, the two hydrogels were cut in the middle, and the two semi-disk-shaped sections of hydrogels with different color were in close contact. the two pieces were left standing at room temperature for 1 h. Finally, this new hydrogel was tested for self-healing and photographed.

### 4.8. Antibacterial Activity

The antibacterial ability of hydrogels was evaluated against *S. aureus* (ATCC 6538) and *E. coli* (ATCC 25922) according to the previous report [69]. Briefly, 0.02 g hydrogel was added into a glass tube with 10 mL LB medium and 10 μL bacterial suspension of *E. coli* (1 × 10^7^ mL^−1^) or *S. aureus* (1 × 10^7^ CFU mL^−1^). The tube without hydrogel was considered as the control group. Then the mixed solution was cultured at 37 °C for 6 h. Finally, a UV-Vis spectrophotometer was used to measure the absorption value of the solution at 600 nm. The antibacterial rates of hydrogels were calculated by the below equation:

$$Antibacterial\;rate=\;\frac{M_{C}-M_{S}}{M_{C}}\;\times 100\%$$

where *M_s_* and *M_c_* are the adsorption of the sample group and the control group, respectively.

### 4.9. Antioxidant Performance

The antioxidant efficiency of the prepared hydrogel was evaluated using a DPPH free radical scavenging assay [70]. Firstly, DPPH was dissolved in ethanol to obtain a concentration of 100 μM. Subsequently, 2 mg Gel 1, Gel 2, and Gel 3 were added to 5 mL of DPPH solution, respectively. The mixture was stirred at room temperature and incubated in the dark for 30 min. Then, a UV-Vis spectrophotometer was used to measure the absorption value of the solution at 517 nm. Finally, the scavenging efficiency of hydrogels on free radicals was calculated through the following formula:

$$DPPH\;scavenging\;rate=\frac{A_{c}\;-\;A_{s}}{A_{c}}\;\times 100\%$$

where *A_c_* and *A_s_* are the absorbance of the blank sample and the sample group, respectively.

### 4.10. Intracellular ROS Scavenging Activity

The antioxidative effect of hydrogels on intracellular ROS was evaluated on RAW cells. Briefly, RAW 264.7 cells were seeded into a 24-well plate and then cocultured with LPS or hydrogel extract for 24 h, respectively. The group without LPS and hydrogel served as the control group. 10 μM DCFH-DA was added into RAW 264.7 cells, and the cells were incubated for 20 min at 37 °C in the dark. The intracellular ROS level was then evaluated using the plate reader. Meanwhile, the fluorescence intensity in the cells was observed by a fluorescence microscope. Lastly, the performance of hydrogels in clearing the intracellular ROS was assessed using flow cytometry [71].

### 4.11. Hemolysis Test

The anticoagulant tube was used to store the fresh blood from male mice. Subsequently, 20 μL blood and 1 mL deionized water were added to the centrifuge tube and set as the positive group. In the negative group, 20 μL blood and 1 mL PBS buffer were added to the centrifuge tube. In the experimental group, 20 μL blood, 1 mL PBS buffer and 20 mg hydrogel were added to the centrifuge tube. All samples were incubated at 37 °C for 1 h and then centrifuged at 3000 rpm for 5 min. Subsequently, a microplate reader was used to detect the absorption value of the solution at a wavelength of 540 nm. Finally, the hemolysis ratio of hydrogel was calculated by the below equation:

$$Hemolysis\;ratio=\;\frac{A_{s}-A_{1}}{A_{0}-A_{1}}\;\times 100\%$$

where *A_s_* was the absorption value of the experiment groups, *A*_0_ and *A*_1_ were the absorption values of the positive group and negative group, respectively.

### 4.12. Cytocompatibility Test

In this experiment, the cytotoxicity of the hydrogel was determined by mouse embryonic fibroblast cells (L929, CCL-1, obtained from Procell Life Science & Technology Co., Ltd., Wuhan, China) through the Cell Counting Kit-8 (CCK-8) method [44]. 1 × 10^5^ cells/mL of L929 cells were seeded on the surface of the hydrogel in a 96-well plate and then incubated in an incubator with 5% CO_2_ at 37 °C for 1, 3, and 5 days, respectively. Cells in the control group were cultured in a medium without the presence of hydrogel samples. Subsequently, the cell viability was determined through a standard CCK-8 assay. Moreover, the morphology of cells after being co-incubated with hydrogel extract for 1, 3, and 5 days was characterized using Live/Dead staining and a laser confocal fluorescence microscope (CLSM, Leica TCS SP5 II, Leica, Vizsla, Germany).

### 4.13. Full-Thickness Skin Wound Model

The promotion effects of hydrogels on full-thickness skin wounds were evaluated according to the previous report [45]. Male mice, aged 7 weeks, were raised under controlled conditions for one week prior to the operation to adapt to the environment. Then, the mice were anesthetized using the intraperitoneal injection method with 50 mg/kg pentobarbital sodium, and a full-thickness wound with a diameter of 15 mm was created on the shaved back using a biopsy punch. Subsequently, various types of hydrogels were used to treat the wounds for 14 days, while the group treated with physiological saline served as the negative control. Each group contained 6 animals. Digital images of wounds on days 0, 4, 7, 10, and 14 were photographed, and the wound area was measured using ImageJ 1.47 software. All in vivo animal experiments have been granted permission by the Animal Ethical Committee of Tongji Medical College, Huazhong University of Science and Technology (HUST), and the ethical approval number is 4441. All animal procedures strictly abided by ARRIVE guidelines and were carried out in accordance with the UK’s Animals (Scientific Procedures) Act 1986 and related guidelines.

### 4.14. Histology and Immunohistochemical Staining

To evaluate the wound healing quality, the healed skin was collected on 4th postoperative day and fixed with paraformaldehyde solution (4%) for further immunohistochemistry staining (CD11b expression), immunofluorescence staining (CD86 and CD206 expression). In addition, the healed skin was also excised on days 7 and 14 for H&E staining and Masson trichrome staining. Histological sections were examined under light microscopy and scored for collagen deposition, the length of the wound distance, and the thickness of epidermal and granulation tissue.

To further evaluate angiogenesis at the wound site, CD31 and α-SMA immunofluorescence staining were performed, while (interleukin-10) IL-10 and (tumor necrosis factor α) TNF-α immunofluorescence staining were used to assess inflammation [72]. Briefly, the wound skin tissues on day 14 were collected and fixed with paraformaldehyde solution (4%). Subsequently, the tissues were embedded in paraffin and sectioned at a thickness of 8 μm. After being fixed with 4% paraformaldehyde at room temperature for 20 min, tissue sections were incubated in a blocking solution containing 6% fetal bovine serum. Then, they were incubated overnight at 4 °C with primary antibodies against CD31, α-SMA, IL-10, and TNF-α. The expression level was analyzed statistically using ImageJ.

### 4.15. Statistical Analysis

The data are expressed as the mean ± standard deviation (SD) of the three measurements, utilizing GraphPad Prism 8 software. Additionally, SPSS software (Version 26.0, SPSS Inc., Chicago, IL, USA) was used to perform a one-way ANOVA analysis to assess the statistical significance of the differences. The significance levels were established at the following thresholds: * *p* < 0.05, ** *p* < 0.01, and *** *p* < 0.001.

## Data Availability

The original contributions presented in this study are included in the article. Further inquiries can be directed to the corresponding author.

## References

[1] Pratsinis H., Mavrogonatou E., Kletsas D.J. (2019). Scarless wound healing: From development to senescence. Adv. Drug Deliv. Rev..

[2] Zhao X., Liang Y., Huang Y., He J., Han Y., Guo B.J.A.F.M. (2020). Physical double-network hydrogel adhesives with rapid shape adaptability, fast self-healing, antioxidant and NIR/pH stimulus-responsiveness for multidrug-resistant bacterial infection and removable wound dressing. Adv. Funct. Mater..

[3] An Y., Lin S., Tan X., Zhu S., Nie F., Zhen Y., Gu L., Zhang C., Wang B., Wei W.J. (2021). Exosomes from adipose-derived stem cells and application to skin wound healing. Cell Prolif..

[4] Zhong S., Zhang Y., Lim C.J. (2010). Tissue scaffolds for skin wound healing and dermal reconstruction. WIREs Nanomed. Nanobiotechnol..

[5] Schäfer M., Werner S. (2008). Oxidative stress in normal and impaired wound repair. Pharmacol. Res..

[6] Wilgus T.A. (2008). Immune cells in the healing skin wound: Influential players at each stage of repair. Pharmacol. Res..

[7] Wilkinson H.N., Hardman M.J. (2020). Wound healing: Cellular mechanisms and pathological outcomes. Open Biol..

[8] Lauer G., Sollberg S., Cole M., Krieg T., Eming S.A., Flamme I., Stürzebecher J., Mann K. (2000). Expression and proteolysis of vascular endothelial growth factor is increased in chronic wounds. J. Investig. Dermatol..

[9] Wallace H.J., Stacey M.C. (1998). Levels of tumor necrosis factor-α (TNF-α) and soluble TNF receptors in chronic venous leg ulcers–correlations to healing status. J. Investig. Dermatol..

[10] Eming S.A., Martin P., Tomic-Canic M. (2014). Wound repair and regeneration: Mechanisms, signaling, and translation. Sci. Transl. Med..

[11] Shen Y.-I., Cho H., Papa A.E., Burke J.A., Chan X.Y., Duh E.J., Gerecht S. (2016). Engineered human vascularized constructs accelerate diabetic wound healing. Biomaterials.

[12] Li Z., Milionis A., Zheng Y., Yee M., Codispoti L., Tan F., Poulikakos D., Yap C.H. (2019). Superhydrophobic hemostatic nanofiber composites for fast clotting and minimal adhesion. Nat. Commun..

[13] Zou Y., Arno M.C., Chen S., Wang T., Gao J., Dove A.P., Du J. (2017). Hydrogel scaffolds for differentiation of adipose-derived stem cells. Chem. Soc. Rev..

[14] Bakadia B.M., Ahmed A.A.Q., Lamboni L., Shi Z., Mukole B.M., Zheng R., Mbang M.P., Zhang B., Gauthier M., Yang G. (2023). Engineering homologous platelet-rich plasma, platelet-rich plasma-derived exosomes, and mesenchymal stem cell-derived exosomes-based dual-crosslinked hydrogels as bioactive diabetic wound dressings. Bioact. Mater..

[15] Rungrod A., Kapanya A., Punyodom W., Molloy R., Meerak J., Somsunan R.J.B. (2021). Synthesis of poly (ε-caprolactone) diacrylate for micelle-cross-linked sodium AMPS hydrogel for use as controlled drug delivery wound dressing. Biomacromolecules.

[16] Liang J., Zhang K., Li J., Su J., Guan F., Li J.J.M. (2022). Injectable protocatechuic acid based composite hydrogel with hemostatic and antioxidant properties for skin regeneration. Mater. Des..

[17] Zhao X., Wu H., Guo B., Dong R., Qiu Y., Ma P.X. (2017). Antibacterial anti-oxidant electroactive injectable hydrogel as self-healing wound dressing with hemostasis and adhesiveness for cutaneous wound healing. Biomaterials.

[18] Farjadian F., Mirkiani S., Ghasemiyeh P., Kafshboran H.R., Mehdi-Alamdarlou S., Raeisi A., Esfandiarinejad R., Soleymani S., Goshtasbi G., Firouzabadi N. (2024). Smart nanogels as promising platform for delivery of drug, gene, and vaccine; therapeutic applications and active targeting mechanism. Eur. Polym. J..

[19] Luo P., Huang R., Wu Y., Liu X., Shan Z., Gong L., Deng S., Liu H., Fang J., Wu S. (2023). Tailoring the multiscale mechanics of tunable decellularized extracellular matrix (dECM) for wound healing through immunomodulation. Bioact. Mater..

[20] Sridharan R., Cameron A.R., Kelly D.J., Kearney C.J., O’Brien F. (2015). Biomaterial based modulation of macrophage polarization: A review and suggested design principles. Mater. Today.

[21] Mao J., Chen L., Cai Z., Qian S., Liu Z., Zhao B., Zhang Y., Sun X., Cui W. (2022). Advanced biomaterials for regulating polarization of macrophages in wound healing. Adv. Funct. Mater..

[22] Kim S.-W., Im G.-B., Jeong G.-J., Baik S., Hyun J., Kim Y.-J., Pang C., Jang Y.C., Bhang S.H. (2021). Delivery of a spheroids-incorporated human dermal fibroblast sheet increases angiogenesis and M2 polarization for wound healing. Biomaterials.

[23] Saoudi M., Badraoui R., Chira A., Saeed M., Bouali N., Elkahoui S., Alam J.M., Kallel C., El Feki A. (2021). The Role of *Allium subhirsutum* L. in the attenuation of dermal wounds by modulating oxidative stress and inflammation in Wistar albino rats. Molecules.

[24] Liu R., Chen S., Huang P., Liu G., Luo P., Li Z., Xiao Y., Chen Z., Chen Z. (2020). Immunomodulation-based strategy for improving soft tissue and metal implant integration and its implications in the development of metal soft tissue materials. Adv. Funct. Mater..

[25] Petkovic M., Sørensen A.E., Leal E.C., Carvalho E., Dalgaard L.T. (2020). Mechanistic actions of microRNAs in diabetic wound healing. Cells.

[26] Abaricia J.O., Shah A.H., Ruzga M.N., Olivares-Navarrete R. (2021). Surface characteristics on commercial dental implants differentially activate macrophages in vitro and in vivo. Clin. Oral Implant. Res..

[27] Atcha H., Jairaman A., Holt J.R., Meli V.S., Nagalla R.R., Veerasubramanian P.K., Brumm K.T., Lim H.E., Othy S., Cahalan M.D. (2021). Mechanically activated ion channel Piezo1 modulates macrophage polarization and stiffness sensing. Nat. Commun..

[28] Feng Z., Su Q., Zhang C., Huang P., Song H., Dong A., Kong D., Wang W. (2020). Bioinspired nanofibrous glycopeptide hydrogel dressing for accelerating wound healing: A cytokine-free, M2-type macrophage polarization approach. Adv. Funct. Mater..

[29] Das S., Majid M., Baker A.B. (2016). Syndecan-4 enhances PDGF-BB activity in diabetic wound healing. Acta Biomater..

[30] Haidari H., Bright R., Strudwick X.L., Garg S., Vasilev K., Cowin A.J., Kopecki Z. (2021). Multifunctional ultrasmall AgNP hydrogel accelerates healing of S. aureus infected wounds. Acta Biomater..

[31] Bonferoni M.C., Caramella C., Catenacci L., Conti B., Dorati R., Ferrari F., Genta I., Modena T., Perteghella S., Rossi S. (2021). Biomaterials for soft tissue repair and regeneration: A focus on Italian research in the field. Pharmaceutics.

[32] Guo W., Ding X., Zhang H., Liu Z., Han Y., Wei Q., Okoro O.V., Shavandi A., Nie L. (2024). Recent advances of chitosan-based hydrogels for skin-wound dressings. Gels.

[33] Zhang K., Huang H., Zhao Y., Zhen Q., Shi D., Chen J., Chen X. (2025). Pullulan dialdehyde cross-linked dual-action adhesive with high adhesion to lung tissue and the capability of pH-responsive drug release. Carbohydr. Polym..

[34] Hu Y., Xiong Y., Zhu Y., Zhou F., Liu X., Chen S., Li Z., Qi S., Chen L. (2023). Copper-epigallocatechin gallate enhances therapeutic effects of 3D-printed dermal scaffolds in mitigating diabetic wound scarring. ACS Appl. Mater. Interfaces.

[35] Ud-Din S., Foden P., Mazhari M., Al-Habba S., Baguneid M., Bulfone-Paus S., McGeorge D., Bayat A. (2019). A double-blind, randomized trial shows the role of zonal priming and direct topical application of epigallocatechin-3-gallate in the modulation of cutaneous scarring in human skin. J. Investig. Dermatol..

[36] Yoon J.Y., Kwon H.H., Min S.U., Thiboutot D.M., Suh D.H. (2013). Epigallocatechin-3-gallate improves acne in humans by modulating intracellular molecular targets and inhibiting P. acnes. J. Investig. Dermatol..

[37] He Z., Luo H., Wang Z., Chen D., Feng Q., Cao X. (2023). Injectable and tissue adhesive EGCG-laden hyaluronic acid hydrogel depot for treating oxidative stress and inflammation. Carbohydr. Polym..

[38] Wang Y., Zhu Z., Lv X., Han B., Jiang Z. (2025). Multifunctional carboxymethyl chitosan-based sponges loaded with epigallocatechin-3-gallate for accelerating wound healing in diabetic rats with full-thickness burns. Carbohydr. Polym..

[39] Huang Y.-W., Zhu Q.-Q., Yang X.-Y., Xu H.-H., Sun B., Wang X.-J., Sheng J. (2019). Wound healing can be improved by (—)-epigallocatechin gallate through targeting Notch in streptozotocin-induced diabetic mice. FASEB J..

[40] Ning Y., Yuan Z., Wang Q., He J., Zhu W., Ren D.n., Wo D. (2024). Epigallocatechin-3-gallate promotes wound healing response in diabetic mice by activating keratinocytes and promoting re-epithelialization. Phytother. Res..

[41] Kumar Srivastava A., Khare P., Kumar Nagar H., Raghuwanshi N., Srivastava R. (2016). Hydroxyproline: A potential biochemical marker and its role in the pathogenesis of different diseases. Curr. Protein Pept. Sci..

[42] Torkaman S., Najafi S.H.M., Ashori A., Mohseni F.A. (2024). Chemoselective modification of chitosan with arginine and hydroxyproline: Development of antibacterial composite films for wound healing applications. Int. J. Biol. Macromol..

[43] Chen L., Cao P., Zhao P., Xu Y., Lv G., Yu D. (2024). Photodynamic-Controllable microneedle composite with Antibacterial, Antioxidant, and angiogenic effects to expedite infected diabetic wound healing. Mater. Des..

[44] Liu S., Zhao Y., Li M., Nie L., Wei Q., Okoro O.V., Jafari H., Wang S., Deng J., Chen J. (2023). Bioactive wound dressing based on decellularized tendon and GelMA with incorporation of PDA-loaded asiaticoside nanoparticles for scarless wound healing. Chem. Eng. J..

[45] Yan L., Liu S., Wang J., Ding X., Zhao Y., Gao N., Xia Z., Li M., Wei Q., Okoro O.V. (2024). Constructing nerve guidance conduit using dECM-doped conductive hydrogel to promote peripheral nerve regeneration. Adv. Funct. Mater..

[46] Ishii S., Kokubo H., Hashimoto K., Imaizumi S., Watanabe M. (2017). Tetra-PEG network containing ionic liquid synthesized via michael addition reaction and its application to polymer actuator. Macromolecules.

[47] Feng Z., Zhang Y., Yang C., Liu X., Huangfu Y., Zhang C., Huang P., Dong A., Liu J., Liu J. (2023). Bioinspired and inflammation-modulatory glycopeptide hydrogels for radiation-induced chronic skin injury repair. Adv. Healthc. Mater..

[48] Rahayu D.P., De Mori A., Yusuf R., Draheim R., Lalatsa A., Roldo M. (2022). Enhancing the antibacterial effect of chitosan to combat orthopaedic implant-associated infections. Carbohydr. Polym..

[49] Queiroz M.F., Teodosio Melo K.R., Sabry D.A., Sassaki G.L., Rocha H.A.O. (2014). Does the use of chitosan contribute to oxalate kidney stone formation?. Mar. Drugs.

[50] Wang Q., Dong Z., Du Y., Kennedy J.F. (2007). Controlled release of ciprofloxacin hydrochloride from chitosan/polyethylene glycol blend films. Carbohydr. Polym..

[51] Tu C.-W., Tsai F.-C., Chen J.-K., Wang H.-P., Lee R.-H., Zhang J., Chen T., Wang C.-C., Huang C.-F. (2020). Preparations of tough and conductive PAMPS/PAA double network hydrogels containing cellulose nanofibers and polypyrroles. Polymers.

[52] Nie L., Li J., Lu G., Wei X., Deng Y., Liu S., Zhong S., Shi Q., Hou R., Sun Y. (2022). Temperature responsive hydrogel for cells encapsulation based on graphene oxide reinforced poly (N-isopropylacrylamide)/hydroxyethyl-chitosan. Mater. Today Commun..

[53] Zhu Y., Hoshi R., Chen S., Yi J., Duan C., Galiano R.D., Zhang H.F., Ameer G.A. (2016). Sustained release of stromal cell derived factor-1 from an antioxidant thermoresponsive hydrogel enhances dermal wound healing in diabetes. J. Control. Release.

[54] Ren X., Han Y., Wang J., Jiang Y., Yi Z., Xu H., Ke Q. (2018). An aligned porous electrospun fibrous membrane with controlled drug delivery–an efficient strategy to accelerate diabetic wound healing with improved angiogenesis. Acta Biomater..

[55] Xu Z., Han S., Gu Z., Wu J. (2020). Advances and impact of antioxidant hydrogel in chronic wound healing. Adv. Healthc. Mater..

[56] Zhang D., Zhou W., Wei B., Wang X., Tang R., Nie J., Wang J. (2015). Carboxyl-modified poly(vinyl alcohol)-crosslinked chitosan hydrogel films for potential wound dressing. Carbohydr. Polym..

[57] Deng Z., Shi F., Zhou Z., Sun F., Sun M.-H., Sun Q., Chen L., Li D., Jiang C.-Y., Zhao R.-Z.J.T. (2019). M1 macrophage mediated increased reactive oxygen species (ROS) influence wound healing via the MAPK signaling in vitro and in vivo. Toxicol. Appl. Pharmacol..

[58] Couture C., Zaniolo K., Carrier P., Lake J., Patenaude J., Germain L., Guérin S.L. (2016). The tissue-engineered human cornea as a model to study expression of matrix metalloproteinases during corneal wound healing. Biomaterials.

[59] Rendra E., Riabov V., Mossel D.M., Sevastyanova T., Harmsen M.C., Kzhyshkowska J. (2019). Reactive oxygen species (ROS) in macrophage activation and function in diabetes. Immunobiology.

[60] Shapouri-Moghaddam A., Mohammadian S., Vazini H., Taghadosi M., Esmaeili S.A., Mardani F., Seifi B., Mohammadi A., Afshari J.T., Sahebkar A. (2018). Macrophage plasticity, polarization, and function in health and disease. J. Cell. Physiol..

[61] Arabpour M., Saghazadeh A., Rezaei N. (2021). Anti-inflammatory and M2 macrophage polarization-promoting effect of mesenchymal stem cell-derived exosomes. Int. Immunopharmacol..

[62] Boniakowski A.E., Kimball A.S., Jacobs B.N., Kunkel S.L., Gallagher K.A. (2017). Macrophage-mediated inflammation in normal and diabetic wound healing. J. Immunol..

[63] Li M., Hou Q., Zhong L., Zhao Y., Fu X. (2021). Macrophage related chronic inflammation in non-healing wounds. Front. Immunol..

[64] Guo Y., Zhang C., Xie B., Xu W., Rao Z., Zhou P., Ma X., Chen J., Cai R., Tao G. (2024). Multifunctional microneedle patch based on metal-phenolic network with photothermal antimicrobial, ROS scavenging, immunomodulatory, and angiogenesis for programmed treatment of diabetic wound healing. ACS Appl. Mater. Interfaces.

[65] Liu K., Liu L., Guo H., Xu R., Liang X., Chen Y., Li H., Fu X., Wang X., Chen H. (2023). Redox modulatory Cu (II)-baicalein microflowers prepared in one step effectively promote therapeutic angiogenesis in diabetic mice. Adv. Healthc. Mater..

[66] Zhang X., Li Y., Ma Z., He D., Li H. (2021). Modulating degradation of sodium alginate/bioglass hydrogel for improving tissue infiltration and promoting wound healing. Bioact. Mater..

[67] Tu Z., Chen M., Wang M., Shao Z., Jiang X., Wang K., Yao Z., Yang S., Zhang X., Gao W. (2021). Engineering bioactive M2 macrophage-polarized anti-inflammatory, antioxidant, and antibacterial scaffolds for rapid angiogenesis and diabetic wound repair. Adv. Funct. Mater..

[68] Zhou J., Wang Z., Yang C., Zhang H., Fareed M.S., He Y., Su J., Wang P., Shen Z., Yan W. (2022). A carrier-free, dual-functional hydrogel constructed of antimicrobial peptide Jelleine-1 and 8Br-cAMP for MRSA infected diabetic wound healing. Acta Biomater..

[69] Ding P., Ding X., Liu X., Lu Y., Zhao Y., Chu Y., Fan L., Nie L. (2024). Injectable, self-healing, antibacterial hydrogel dressing based on oxidized dextran and sialic acid substituted chitosan with incorporation of tannic acid. Eur. Polym. J..

[70] Nie L., Wei Q., Sun M., Ding P., Wang L., Sun Y., Ding X., Okoro O.V., Jiang G., Shavandi A. (2023). Injectable, self-healing, transparent, and antibacterial hydrogels based on chitosan and dextran for wound dressings. Int. J. Biol. Macromol..

[71] Zhang X., Feng J., Feng W., Xu B., Zhang K., Ma G., Li Y., Yang M., Xu F.-J. (2022). Glycosaminoglycan-based hydrogel delivery system regulates the wound microenvironment to rescue chronic wound healing. ACS Appl. Mater. Interfaces.

[72] Zhang B., He J., Shi M., Liang Y., Guo B. (2020). Injectable self-healing supramolecular hydrogels with conductivity and photo-thermal antibacterial activity to enhance complete skin regeneration. Chem. Eng. J..

